# Retrospective case-series of *Paecilomyces lilacinus* ocular mycoses in Queensland, Australia

**DOI:** 10.1186/s13104-015-1591-0

**Published:** 2015-10-31

**Authors:** Liam Daniel Turner, Diana Conrad

**Affiliations:** Ophthalmology Department, The Royal Brisbane and Women’s Hospital, 10/87 Hampstead Road, Highgate Hill, Brisbane, QLD 4101 Australia

**Keywords:** Fungal infection, Paecilomyces, Voriconazole

## Abstract

**Background:**

The purpose of this study was to report: (1) the varying presentation of Paecilomyces ocular infections arising in Queensland; (2) the significance of immunosuppression as a primary determinant of disease; (3) the outcomes of voriconazole use; and (4) the ongoing need for both surgical and medical management of this devastating fungal infection.

**Methods:**

A retrospective case series of 21 culture proven individuals participated in this series and were identified via a review of the pathology reporting system utilized in the Queensland public health system. All culture proven individuals were subjected to a systematic chart review.

**Results:**

The primary risk factor for Paecilomyces lilacinus infection is immunosuppression with 81.25 % of individuals being on some form of immunosuppression (i.e. systemic or topical). Of the cases 71.43 % had an intact epithelial surface at the time of diagnosis, and 76 % had no previous ocular history. The final visual outcomes were nine cases with HM vision or worse, three cases with 6/48–6/60 vision, three cases 6/12–6/24, and six cases with 6/12 vision or better. Despite voriconazole use rates of greater than 80 %, protracted and poor treatment outcomes continue to be commonplace.

**Conclusions:**

Paecilomyces lilacinus is a filamentous fungus that has a predilection for immunosuppressed individuals. Despite in vitro and case reports demonstrating the effectiveness of voriconazole poor outcomes continue to be seen.

## Background

Paecilomyces is a filamentous saprophytic fungus that is found worldwide in soil, and as a contaminant in air and water. The fungus is typically resistant to multiple fungicidal agents, and was once considered primarily as a contaminate in culture due to its inherent resistance to available commercial sterile techniques [1, 2]. It is also found in fertilizers due to its bionematicidal effectiveness against nematodes, which threaten commercial vegetation [3]. The species include: *Paecilomyces lilacinus*, *Paecilomyces variotti*, *Paecilomyces marquadnii* and *Paecilomyces javanicus*, the former two being the most common cause of disease in humans [4–7]. Infection with Paecilomyces species is most common in the setting of immunosuppression [8–11], both topical and systemic, with rates of 76 % corticosteroid use in patients with ocular mycoses prior to diagnosis [12]. Cases of Paecilomyces infection predominantly include ocular mycoses and mycoses of cutaneous or subcutaneous tissues [7]. Paecilomyces has been shown to cause disease elsewhere in the body, but its predilection for the ocular surface and skin is thought to be due to a thermal intolerance of the fungus, with the optimum temperature for growth and sporulation, somewhere in the vicinity of 20–25 °C [13].

Ocular infection with *Paecilomyces lilacinus* has previously been reported to occur in the setting of chronic keratopathy, after previous ocular surgery, following corneal trauma, or with the use of soft contact lenses [12, 14]. The literature with respect to previous ocular surgery is somewhat skewed, with a large number of cases being reported in the early 1980s, occurring in the presence of contaminated intraocular lens implantation [15, 16]. A few cases exist within the literature, demonstrating the occurrence of *Paecilomyces lilacinus* infection in the setting of an intact epithelial surface [17–22]. These cases initially presented with presumed immune-mediated scleritis [21], nodular episcleritis [18], acute anterior uveitis [19, 20], endophthalmitis [20], and corneal stromal or endothelial inflammation [17]. It has been postulated that an endogenous spread of the organism may be the underlying source of infection in these cases with an intact epithelium [17, 20, 22, 23]. However, few reports exist that identify *Paecilomyces lilacinus* within the systemic vasculature. *Paecilomyces lilacinus* has been identified in blood cultures, primarily in the presence of indwelling venous catheters, which subsequently became sterile after removal of the device [8, 24.]

A review of previous cases identified within Queensland, Australia was conducted to demonstrate: [1] the varying presentation of Paecilomyces ocular infections arising in Queensland; [2] the significance of immunosuppression as a primary determinant of disease; [3] the outcomes of voriconazole use; [4] the ongoing need for both surgical and medical management of this devastating fungal infection; and [5] the need for protracted treatment.

## Methods

A retrospective multi-centre case series was conducted of all culture proven cases of ocular *Paecilomyces lilacinus* occurring within Queensland Health between 2000 and 2012. Ethics approval was gained from the Queensland Health Central Health and Medical Research Human Ethics Committee. Research adhered to the tenets of the Declaration of Helsinki. A search was undertaken of the electronic pathology system utilized by Queensland Health, namely Auslab and Auscare. A multi-centre study was conducted with cases coming from the two major referral centers within Queensland, The Princess Alexandra Hospital and The Royal Brisbane and Women’s Hospital. Search terms were *Paecilomyces lilacinus* and Paecilomyces species. Consent was obtained from participants for treatment undertaken.

Within the database a total of 135 cases of *Paecilomyces lilacinus* infection were identified. All non-ocular Paecilomyces infections were excluded from this study, providing a total of 21 cases of ocular *Paecilomyces lilacinus* infection with samples coming from corneal tissue, corneal scrapes, aqueous and vitreous biopsy. Specimens were transported in sterile containers or in syringes in the case of fluid specimens. Specimens were inoculated on Sabouraud’s agar at 25 °C for up to 1 month. Identification was performed via phenotypic methods at local facilities. Some of these cases have been previously documented within the literature [18, 22]. Identified cases, were subjected to a systematic chart review. Information obtained during the review, included: name, record unit number, age, gender, resident location, specimen type, date of collection, date of presentation, initial diagnosis, actual diagnosis, risk factors, initial treatment and management prior to recognition of fungal infection, elapsed time before positive diagnosis, continued treatment both medical and surgical, outcome of management (i.e. resolution of infection, enucleation, phthisical eye), final visual acuity, duration of follow-up and previous ocular history.

Data obtained from the chart review were analysed, formulating simple descriptive statistics, utilising RCommander Version 2.15.2 GUI 1.53.

## Results

The case series consisted of 6 females and 15 males with a mean age of 52.48 years (SD = 17.51; range = 19.0–76.0) (Refer to Table 1—cases). The average distance from Brisbane Central Business District (CBD) was 340 km (SD = 578.94, range = 12.6–1755.0). The average duration of follow-up from the time of initial diagnosis to last review was 28.19 months (SD = 38.52; range = 1.0–144; median = 7 months). Sixteen (76 %) of the patients had no previous history of ocular disease or surgery, with *Paecilomyces lilacinus* infection being the initial presenting problem for all these cases. Of the remaining five cases, two had a history of myopia and soft contact lens wear, two were bilateral pseudophakic, with one also having had a previous retinal detachment managed with scleral buckle, and one had a previous history of scleritis.

**Table 1:** Cases

Case/identified (year)	Site	Presentation	Immunosuppression/risk factor	Specimen	Past ocular history	Comorbidities	Treatment
1 (2000)	PAH	Hypopyon	Suspected trauma + PO + topical steroids	Corneal biopsy/aqueous fluid	6 months prior biopsy of sclera taken for lesion—showed chronic inflammation lead to treatment with topical and PO steroids	Nil	AC tap × 3 + corneal biopsy. IV amphotericin + topical natomycin. Intracameral amphotericin × 4. Enucleation
2 (2001)	RBWH	Endophthalmitis	PO prednisolone, cyclophosamide, methotrexate	Vitreous fluid	11 months prior treated for anterior necrotizing scleritis. Subsequently developed endophthalmitis	Nil	Vitreous tap performed. Commenced topical natomycin, PO itraconazole, and PPV/AC washout/intravitreal amphotericin. Intravitreal × 4 amphotericin + IV. PO voriconazole. Enucleation
3 (2001)	RBWH	AC and anterior vitreous inflammation	PO steroids	Vitreous fluid	Nil	Ulcerative colitis	Presumed inflammatory secondary to underlying autoimmune illness. Treated with 2 weeks of topical steroids prior to referral. PPV/vitreous biopsy/intravitreal amphotericin/ceftazadime/vancomycin + IV of all three. 2 × further intravitreal amphotericin. Repeat PPV/intravitreal amphotericin + vancomycin
4 (2002)	PAH	AC and anterior vitreous inflammation	Topical + PO steroids. Park tractor mower operator	Vitreous fluid	Nil	Nil	AC tap performed. Commenced IV, intravitreal and topical amphotericin. PPV/lensectomy/intravitreal amphotericin. PO voriconazole. Repeat PPV/Intravitreal amphotericin. PK/iridectomy/washout/amphotericin
5 (2002)	PAH	Deep stromal infiltrate	Vegetative matter versus eye + topical steroids	Aqueous fluid	Nil	Nil	FB versus eye. Treated with topical steroids + antibiotics. Proceeded to develop deep stromal infiltrate. Presumed fungal keratitis. Corneal scrape + AC tap. Commenced topical natamycin + amphotericin and PO itraconazole. Multiple repeat cultures (corneal biopsy × 2). IV amphotericin + PO voriconazole. PK
6 (2002)	PAH	Endothelial plaque 2.4 mm × 1.8 mm	Nil	Vitreous fluid	Nil	Nil	Aqueous tap showed fungal hyphae. Natamycin topical, itraconazole PO + amphotericin IV commenced. PK/lamellar sclerotomy/iridectomy. Recurrence lead to PPV/iridectomy/lensectomy/intravitreal amphotericin. Amphotericin topical and voriconazole PO
7 (2002)	PAH	Anterior chamber reaction	Nil	Vitreous fluid	Nil	Nil	Red painful eye plus AC reaction. Steroid challenge. Deteriorated to endothelial change. Corneal biopsy. PK. IV amphotericin. PPV/lensectomy/iridectomy/intravitreal amphotericin. Repeat PK. PO voriconazole
8 (2004)	PAH	Interstitial keratitis	Metal worker + topical and PO steroids	Corneal biopsy	Nil	Nil	AC tap. Topical natomycin + PO itraconazole. Corneal biopsy. Topical voriconazole. PK/iridectomy. Topical + PO voriconazole
9 (2004)	RBWH	Keratitis	Vegetative matter versus eye	Corneal scrape	Nil	Nil	Corneal scrape. Treated with topical gentamicin and ceftazidime. Improved without antifungals
10 (2004)	PAH	Keratoscleritis	Nil	Corneal biopsy	Nil	Nil	Topical natomycin. PO fluconazole + voriconazole. Corneoscleral graft/anterior vitrectomy
11 (2006)	PAH	Deep stromal infiltrate	Topical steroids	Aqueous fluid	Seen by multiple ophthalmologists. Red, painful eye treated with topical steroids	Nil	Developed deep stromal infiltrate with no epithelial defect. Corneal scrape. Commenced on PO and topical voriconazole. AC Tap. Repeat Corneal scrape. Corneal biopsy 3 mm. Corneoscleral graft/AC voriconazole. Topical natomycin. Phaco/IOL
12 (2008)	PAH	Scleritis	Topical + PO prednisolone + PO cyclophosamide	Corneal biopsy	Bisphosphonate scleritis	Osteoporosis	Voriconazole PO + topical + corneal biopsy and aqueous tap. Amphotericin intracameral. PK + intracameral amphotericin. 3 × PKs, 1 corneoscleral graft/iridectomy/lensectomy. 15 × intracameral voriconazole. Enucleation
13 (2008)	PAH	FB (grass tree) to eye	Topical steroids	Corneal biopsy	Bilateral pseudophakic	Nil	Initially treated with topical ofloxacin post FB (improved). Topical steroids commenced lead to worsening of symptoms. AC tap, repeat AC tap and corneal scrape. Voriconazole PO, topical and intracameral. PK + intracameral voriconazole. Repeat PK + IV, intracameral, intravitreal amphotericin, PO terbinafine and topical natomycin added
14 (2009)	RBWH	Vitritis? Toxoplasmosis	Nil	Vitreous fluid	Nil	Nil	Vitreous Tap × 2. Second showed filamentous fungi. Topical, intravitreal × 2, PO voriconazole + PO terbinafine. PPV/intravitreal amphotericin
15 (2009)	RBWH	Vitritis? Toxoplasmosis	Works with organic matter	Vitreous/aqueous fluid	Bilateral pseudophakic. Right RRD (Buckled). 4 months prior diagnosed with Toxoplasmosis. Treated with PO pyrimethamine + sulfadiazine	Nil	Relapse lead to vitreous tap + intravitreal ceftazadime/vancomycin/triamcinolone. 5 × intravitreal, topical + PO voriconazole. 2 × PPV
16 (2009)	RBWH	Presumed contact lens associated microbial keratitis	Contact lens wearer	Corneal scrape	Myope	Nil	Corneal scrape performed. Fungal hyphae identified. Topical voriconazole
17 (2009)	PAH	Pseudomonas contact lens associated keratitis	Contact lens wearer, topical steroids	Corneal scrape	2-month prior treated successfully for culture positive pseudomonas contact lens associated microbial keratitis. Topical steroids commenced lead to worsening of symptoms	Nil	Corneal scrape + AC tap. Fungal elements identified. Topical natamycin + voriconazole topical and PO. PK + intracameral voriconazole. 7 × intracameral voriconazole. PO terbinafine + posaconazole (due to LFT derangement by voriconazole). Glaucoma—cyclodiode × 2. Cataract extraction
18 (2009)	PAH	Acute anterior uveitis	Sulfasalazine, azathioprine	Vitreous fluid	Nodular episcleritis treated 6 month earlier with topical FML and Prednefrin forte	Crohn’s disease	Presented with AC inflammation treated with topical Prednefrin forte. Did not improve lead to vitreous tap. Fungal elements seen on tap. PO voriconazole. PPV + intravitreal voriconazole, amphotericin. Went onto have Phaco/IOL
19 (2010)	RBWH	Keratoscleritis	PO prednisolone + topical steroid. Tropical fruit farmer	Corneal biopsy	4 months prior treated with topical and PO steroid + PO NSAID for nodular scleritis	Nil	Progressive disease leading to corneal involvement. Infection suspected. Corneal biopsy. PO, topical voriconazole commenced. PK. Recurrence. Corneoscleral graft. Phaco/wash‐out/intracameral voriconazole. Iridectomy/PPV/intravitreal voriconazole + amphotericin. Patient underwent weekly then 2nd weekly intravitreal voriconazole for 6 months as unable to tolerate PO voriconazole. Sutured IOL with artificial iris
20 (2012)	RBWH	Keratitis	Topical steroid	Corneal biopsy/aqueous fluid	2 months prior treated for AAU. Failed to attend follow‐up	Nil	Represented with AC inflammation with deep corneal involvement. Biopsy + AC tap. PO + topical voriconazole. 8 × intracameral voriconazole. Topical natamycin
21 (2012)	RBWH	Endophthalmitis	PO + topical steroid + cyclophosamide	Aqueous fluid	6 months prior treated for diffuse anterior scleritis	Type 2 diabetes	Developed dense AC reaction. AC tap performed. PO + topical + intracameral × 7 voriconazole, IV amphotericin. Enucleation

The primary risk factor for Paecilomyces infection was immunosuppression, with 50 % of patients being on systemic immunosuppression (i.e. corticosteroids, cyclophosamide, azathioprine, sulfasalazine and methotrexate), 31.25 % on topical immunosuppression (i.e. dexamethasone, prednisolone, fluoromethalone), 12.5 % having exposure to organic material and 12.5 % wearing soft contact lenses (one of the patients who wore soft contacts was also treated with topical steroids). Initial presenting diagnoses for patients included: 6 with scleritis/episcleritis, 1 acute anterior uveitis, 2 with presumed toxoplasmosis, 3 with foreign body induced keratitis, 2 with contact lens associated keratitis, 1 with herpetic interstitial keratitis, 1 with endogenous endophthalmitis, and 1 with an endothelial plaque with an uncertain diagnosis, and 3 patients had no clear diagnosis at presentation.

The time to positive diagnosis of *Paecilomyces lilacinus* infection was on average 12.75 days (SD = 9.51; range = 2–40). With final diagnoses for patients including: 6 fungal endophthalmitis (28.57 %), 9 fungal keratitis (42.86 %), 4 fungal keratoscleritis (19.05 %), 1 fungal keratitis leading to endophthalmitis (4.76 %), and 1 fungal keratoscleritis leading to endophthalmitis (4.76 %). Of these cases, 15 had an intact epithelial surface (71.43 %) and 5 had a compromised epithelium (23.81 %). Medical management consisted of voriconazole, which was prescribed orally in 17 cases, followed by amphotericin, which was given intravenously in 7 cases (Refer to Table 2—antifungal agent use). Some individuals required greater than 15 intravitreal injections of voriconazole in order to assist in resolution of the infection. One case of fungal keratitis, secondary to a foreign body, resolved without antifungal agents, solely with the use of ceftazidime and gentamicin. Of the 21 cases, 18 cases (85.7 %) required surgical intervention in order to help resolve the infection. Surgical intervention included, 8 cases, which underwent penetrating keratoplasty and 9 cases, which received a pars plana vitrectomy (PPV). Of those receiving a penetrating keratoplasty, 5 required at least a second penetrating keratoplasty, with one individual having a total of 4 grafts. Of the cases receiving PPV, 4 cases of the group went onto have a second PPV (Refer to Table 3—surgical intervention). The final outcome for treatment, included 14 cases with resolution of infection (66.67 %), 4 cases with enucleation (19.05 %) and 3 cases with phthisis (14.29 %). The final visual outcomes were 9 cases with HM vision or worse, 3 cases with 6/48–6/60 vision, 3 cases 6/12–6/24, and 6 cases with 6/12 vision or better (Refer to Table 4—visual outcomes).

**Table 2:** Antifungal agent use

Anti-fungal	No. of cases
Amphotericin	
Intravenous	7
Topical	2
Intracameral	2
Intravitreal	7
Voriconazole	
Oral	17
Topical	11
Intracameral	6
Intravitreal	3
Natamycin topical	8
Itraconazole oral	3
Terbinafine oral	2
Posaconazole oral	1
Fluconazole oral	1

**Table 3:** Surgical intervention

Surgical intervention	No. of cases (%)
Penetrating keratoplasty	8 (38)
Corneoscleral graft	4 (19)
Iridectomy	5 (24)
Lensectomy	5 (24)
Pars plan vitrectomy (PPV)	9 (43)
Enucleation	4 (19)

**Table 4:** Visual outcomes

Case	Visual acuity at presentation	Final visual acuity
1	6/36 PH 6/12	Enucleation
2	HM	Enucleation
3	HM	NPL
4	HM	NPL
5	HM	6/60
6	6/28 PH 6/12	NPL
7	6/60 PH 6/36	HM
8	HM	6/24 PH 6/9
9	6/9	6/6
10	6/120	6/60
11	Not recorded	CF PH 6/12
12	HM	Enucleation
13	6/24	6/24 PH 6/19
14	HM	6/24 PH 6/15
15	6/24 PH 6/20	PL
16	6/24 PH 6/10	6/7.5
17	HM	6/120 PH 6/48
18	6/18 PH 6/9	6/6
19	6/36 PH 6/9	6/24 PH 6/18
20	6/20 PH 6/10	6/10 PH 6/7.5
21	PL	Enucleation

## Discussion

Australia appears to have a high number of *Paecilomyces lilacinus* ocular infections in comparison to other parts of the world [7, 22]. Infection has typically been reported to arise in individuals with chronic ocular disease, contact lens use or where the integrity of the eye has been disturbed (i.e. trauma, surgery) [12, 14]. In our study, we found that the majority of cases of infection arose in patients who had an intact epithelial surface and no previous ocular history, with 76 and 71.43 %, respectively. This is markedly different from one of the largest case series currently within the literature, which showed that only 5 of 17 individuals (29.4 %) had no apparent precipitating factor [12], but in line with a recent published case series from Queensland, Australia which also showed the majority of patients also had no specific inciting cause [16, 22].

Previous case reports do exist in the literature highlighting the absence of epithelial breakdown and subsequent *Paecilomyces lilacinus* infection [17–23, 25], including a case of a suspected immune-mediated scleritis and another of acute anterior uveitis [19, 21]. Other cases have also been reported, with a number included in this current review [18, 22]. The authors do not postulate as to the apparent mechanism of the infection, other than to state that it is most likely associated with systemic immunosuppression, previous history of scleritis, diabetes or a previous biopsy that may have contributed to the evolution of the disease [21]. It has been suggested that Paecilomyces may be able to penetrate through an undisturbed epithelial surface or through micro-defects not visible to the naked eye [25]. Some researchers have also suggested that it may spread endogenously, even though few blood culture positive cases have been reported previously [17, 23]. Blood culture positive Paecilomyces typically occurs in association with intravascular prostheses [8, 24]. Furthermore, studies demonstrating the ability of Paecilomyces to actively infect animal and human corneas have either involved inoculation directly into the stroma [26] or via scarification of the cornea [12]. We have been unable to identify a study that has attempted to demonstrate whether Paecilomyces can actively penetrate an intact epithelium.

Immunosuppression is a significant determinant in the pathogenesis of paecilomyces infections. Previous reports highlighted the presence of immunosuppression in 76 % of cases of paecilomyces keratitis prior to diagnosis [12]. Murine models, with immunosuppressed mice (i.e. where their drinking water contained dexamethasone [10] or intraperitoneal cyclophosamide [27]), versus immunocompetent mice, show an inability of paecilomyces to cause disease in the absence of immunosuppression. The mortality in immunosuppressed murine models is incredibly high with one hundred percent of mice succumbing to fungaemia 35–45 days post inoculation [10]. These laboratory models demonstrate the importance of immunosuppression as an important factor in the causal pathway of disease. In our study we found that 81.25 % of individuals were on some form of immunosuppression prior to diagnosis, either in the form of systemic or topical immunosuppression, further providing weight to the importance of immunosuppression as a risk factor.

Infection with *Paecilomyces lilacinus*, is notoriously resistant to available antifungal preparations. Clinical efficacy has been demonstrated for the use of voriconazole monotherapy [18, 26] and in combination with terbinafine [1, 5, 7, 28, 29]. In vitro evidence also exists for the efficacy for posaconazole and ravuconazole [7], and one of the patients in the series did receive oral posaconazole, due to deranged liver functions as a result of oral voriconazole therapy. A recent case report also highlights the clinical efficacy of posaconazole in paecilomyces infection [30]. Voriconazole, a triazole antifungal, which inhibits fungal cytochrome P-450 mediated 14 α-lanosterol demethylation, a necessary step in ergosterol synthesis. This leads to a loss of ergosterol, which is an essential component of the fungal cell wall. In-vitro minimum inhibitory concentrations (MICs) for voriconazole range from 0.12 to 4.0 mg/L [7]. Numerous cases, within the literature have demonstrated the effective use of oral, topical, intravitreal and intracameral use of voriconazole for ocular Paecilomyces infection [5, 7, 12, 18, 29, 31–35]. Eighty percent of cases within our study were treated with voriconazole, at least with an oral preparation. Despite, the higher rate of voriconazole use within the study, 19 % of patients still went onto have an enucleation, which was higher than that previously reported, predominantly in the absence of voriconazole use (5 %) [12]. Furthermore, a significant number of patients in our series required combined surgical intervention in order to assist in resolution of the infection, with 85 % requiring either a penetrating keratoplasty, pars plana vitrectomy or enucleation. This is also greater than that previously demonstrated by Yuan and colleagues, but equivalent to their literature review of current cases in their article [12]. It is therefore suspected that even with the increased susceptibility of *Paecilomyces lilacinus* to voriconazole treatment that combined surgical and medical management will remain the norm [22]. In addition, of the patient’s undergoing surgical intervention, 66 % required repeated surgical intervention, in combination with protracted medical management with voriconazole. Individuals received a minimum of 3 months oral voriconazole, with topical, intracameral or intravitreal voriconazole use dictated on a case-by-case basis.

## Conclusion

We believe that *Paecilomyces lilacinus* ocular infections require persistent and aggressive treatment, with combined surgical and medical management, which patients may not be willing to undertake. Outcomes of Paecilomyces ocular infection should be clearly discussed with patients inflicted with this devastating organism.
